# HIV testing history and access to treatment among migrants living with HIV in Europe

**DOI:** 10.1002/jia2.25123

**Published:** 2018-07-19

**Authors:** Ibidun Fakoya, Débora Álvarez‐Del Arco, Susana Monge, Andrew J Copas, Anne‐Francoise Gennotte, Alain Volny‐Anne, Claudia Wengenroth, Giota Touloumi, Maria Prins, Henrique Barros, Katharine EA Darling, Tullio Prestileo, Julia Del Amo, Fiona M Burns, the aMASE Study Team, A Aerssens, M Aguado, B Alimi, O Anagnostou, J Anderson, A Antoniadou, M Arando, MJ Barberà, A Barthélemy, J Belda‐Ibáñez, B Bertisch, J Bil, JR Blanco, K Block, C Boesecke, M Boura, J Burgos, J Cabo, E Calabuig, L Campbell, O Cardoso, W Claudia, N Clumeck, A Colucci, S Corrao, S Cuellar, J Cunha, G Daikos, K Darling, J del Romero, P Dellot, P Domingo, F Dronda, F Ebeling, A Engelhardt, B Engler, J Farrell, J Fehr, M Feijó, E Fernández, E Fernández García, T Fernandez, AL Fortes, J Fox, P Garcia de Olalla, F García, P Gargalianos‐Kakolyris, I Germano, G Gilleran, R Gilson, S Goepel, HA Gogos, JL Gómez Sirvent, I Gountas, A Gregg, F Gutiérrez, MM Gutierrez, I Hermans, JA Iribarren, H Knobel, L Koulai, S Kourkounti, C La Morté, T LeCompte, B Ledergerber, L Leonidou, MC Ligero, G Lindergard, S Lino, MJ Lopes, A Lopez Lirola, M Louhenapessy, G Lourida, AM Luzi, F Maltez, L Manirankunda, A Martín‐Pérez, L Martins, M Masía, MG Mateu, P Meireles, A Mendes, S Metallidis, S Mguni, A Milinkovic, JM Miró, K Mohrmann, M Montero, T Mouhebati, M Moutschen, M Müller, C Murphy, C Nöstlinger, I Ocaña, S Okumu‐Fransche, G Onwuchekwa, JE Ospina, D Otiko, P Pacheco, R Palacios, V Paparizos, V Papastamopoulos, V Paredes, N Patel, T Pellicer, A Peña, N Petrosillo, A Pinheiro, J Poças, A Portillo, F Post, F Prestileo, P Prins, K Protopapas, M Psichogiou, F Pulido, J Rebollo, A Ribeirinho, I Río, M Robau, JK Rockstroh, E Rodrigues, M Rodríguez, C Sajani, M Salavert, R Salman, N Sanz, G Schuettfort, G Schüttfort, C Schwarze‐ Zander, R Serrão, D Silva, V Silva, P Silverio, A Skoutelis, C Staehelin, C Stephan, C Stretton, F Styles, AF Sutre, S Taylor, B Teixeira, C Thierfelder, O Tsachouridou, K Tudor, E Valadas, M van Frankenhuijsen, M Vázquez, M Velasco Arribas, M Vera, P Vinciana, N Voudouri, JC Wasmuth, E Wilkins, L Young, S Yurdakul, T Zafra Espinosa, W Zuilhof, F Zuure

**Affiliations:** ^1^ Institute for Global Health University College London London United Kingdom; ^2^ National Centre of Epidemiology Instituto de Salud Carlos III Madrid Spain; ^3^ Department of Health and Socio‐medical Sciences University of Alcalá Alcalá de Henares Madrid; ^4^ Department of Infectious Diseases CHU Saint‐Pierre Brussels Belgium; ^5^ European AIDS Treatment Group; ^6^ HIV Centre Frankfurt Germany; ^7^ Department of Hygiene, Epidemiology& Medical Statistics Medical School National and Kapodistrian University of Athens Athens Greece; ^8^ Academic Medical Centre University of Amsterdam Amsterdam the Netherlands; ^9^ Public Health Service of Amsterdam the Netherlands; ^10^ Faculty of Medicine University of Porto Porto Portugal; ^11^ Lausanne University Hospital Lausanne Switzerland; ^12^ Unit for Infectious Diseases and Assistance, Coordination and Territorial Integration for Migrants’ Emergency Civico‐ Benfratelli Hospital Palermo Italy; ^13^ Royal Free London NHS Foundation Trust London England

**Keywords:** HIV, migrants, HIV serodiagnosis, primary healthcare, health services accessibility

## Abstract

**Introduction:**

Migrants are overrepresented in the European HIV epidemic. We aimed to understand the barriers and facilitators to HIV testing and current treatment and healthcare needs of migrants living with HIV in Europe.

**Methods:**

A cross‐sectional study was conducted in 57 HIV clinics in nine countries (Belgium, Germany, Greece, Italy, The Netherlands, Portugal, Spain, Switzerland and United Kingdom), July 2013 to July 2015. HIV‐positive patients were eligible for inclusion if they were as follows: 18 years or older; foreign‐born residents and diagnosed within five years of recruitment. Questionnaires were completed electronically in one of 15 languages and linked to clinical records. Primary outcomes were access to primary care and previous negative HIV test. Data were analysed using random effects logistic regression. Outcomes of interest are presented for women, heterosexual men and gay/bisexual men.

**Results:**

A total of 2093 respondents (658 women, 446 heterosexual men and 989 gay/bisexual men) were included. The prevalence of a previous negative HIV test was 46.7%, 43.4% and 82.0% for women, heterosexual and gay/bisexual men respectively. In multivariable analysis previous testing was positively associated with: receipt of post‐migration antenatal care among women, permanent residency among heterosexual men and identifying as gay rather than bisexual among gay/bisexual men. Access to primary care was found to be high (>83%) in all groups and was strongly associated with country of residence. Late diagnosis was common for women and heterosexual men (60.8% and 67.1%, respectively) despite utilization of health services prior to diagnosis. Across all groups almost three‐quarters of people on antiretrovirals had an HIV viral load <50 copies/mL.

**Conclusions:**

Migrants access healthcare in Europe and while many migrants had previously tested for HIV, that they went on to test positive at a later date suggests that opportunities for HIV prevention are being missed. Expansion of testing beyond sexual health and antenatal settings is still required and testing opportunities should be linked with combination prevention measures such as access to PrEP and treatment as prevention.

## 1 Introduction

The HIV epidemic in Europe is characterized by a disproportionate number of infections among migrants. Although foreign citizens only made up 7% of the population of the European Union (EU) in 2014 1, an estimated 37% of new HIV diagnoses in the EU/European Economic Area (EEA) in 2015 were among migrants 2. Late diagnosis is a feature of the HIV epidemic among migrants; European surveillance data indicate that some migrant groups are more than twice as likely to be diagnosed late than nonmigrants 3.

There are several reasons why migrants are at increased risk of HIV in Europe. Migrants from countries with a generalized HIV epidemic obviously have an increased risk of acquiring HIV before migration, but this risk remains as individuals migrate into, and become sexually active within, migrant communities where the HIV prevalence is higher than the receiving country population 4. Estimates of post‐migration HIV acquisition are as high as 62% in some populations 5. In addition, social inequalities associated with migration (e.g. low income, unemployment, poor housing) 6, HIV‐related stigma and discrimination, and changes in sexual behaviour may increase the risk of late diagnosis or HIV infection 7, 8, 9, 10.

Controlling the HIV epidemic within Europe is dependent on ensuring that migrants have prompt access to HIV testing, antiretroviral therapy (ART) and ongoing healthcare 5, 11, 12. Migrant populations are, of course, heterogeneous making it difficult for policymakers and HIV prevention specialists to provide interventions and services targeted at specific migrant sub‐groups. Often research in this area focuses on one migrant population (e.g. Central and Eastern Europeans 13) or migrants in one country 14. Most of the available research has been conducted with heterosexual migrants from Sub‐Saharan Africa 15. While this reflects the global HIV epidemic, the heterogeneity of migrants living with HIV in Europe 3 rationalizes researching other population groups, particularly migrant gay and bisexual men. In this study we present the results of the first collaborative European survey examining the key socio‐demographic, behavioural and structural factors associated with HIV testing and primary care utilization among migrants living with HIV in Europe. We examine how these factors differ across gender‐related group and present recommendations for targeted health promotion and intervention development.

## 2 Methods

### 2.1 Study design

Full details of the methods used in the aMASE (advancing Migrant Access to health Services in Europe) Study have been described elsewhere 16. A convenience sample was recruited within 57 clinics across the EuroCoord European Network of Excellence on HIV Research (http://www.eurocoord.net). Data collection took place in nine countries (Belgium, Germany, Greece, Italy, The Netherlands, Portugal, Spain, Switzerland and the United Kingdom) between July 2013 and July 2015. Patients were eligible for inclusion if they were (1) HIV positive, (2) aged 18 years and over, (3) foreign‐born and resident in the country of recruitment for six months or more, (4) diagnosed within five years of the study date and (5) able to complete, either alone or supported, a computer‐assisted self or personal interview in any one of the 15 languages available (Amharic, Arabic, Dutch, English, French, German, Greek, Italian, Polish, Portuguese, Russian, Turkish, Tigrinya, Spanish and Somali). In Switzerland, migrants from neighbouring Austria, France, Germany and Italy were excluded.

Eligible participants were identified through clinic records and asked to participate by clinicians or recruitment researchers. Most participants completed within‐questionnaire “tick box” informed consent; participants in Belgium, Switzerland, Greece and Germany completed additional separate consent forms required by local research ethics committees.

The survey instrument was developed by an expert panel made up of experienced epidemiologists and community representatives and covered: socio‐demographic characteristics (including migration history); sexual and HIV risk behaviour; health service use and experiences of living with HIV. Questionnaires were matched to clinical records (CD4 cell counts, viral loads, viral clades, HIV testing history, co‐infections, AIDS‐defining illnesses, treatment initiation) using a unique study number.

The target sample size was 2000 participants (1000 men and 1000 women) from all clinics. Participants were recruited from a minimum of two clinics in each of the nine countries, with each clinic forming a discrete cluster. We assumed the intra‐cluster correlation would be relatively weak (e.g. 0.005), at least after adjustment for country of residence and other variables selected into our statistical regression models. Assuming an average cluster size of 50 participants, the design effect for the study is 1.25 and hence an overall effective sample size of approximately 1600. With this effective sample size outcomes within each gender are estimated to be within 3.5% across Europe based on a 95% confidence interval, and to be within 10% for each country (even if the assumed underlying prevalence is 50%, which would minimize precision).

### 2.2 Ethics

Ethical approval was obtained separately in each participating country. See Additional file 1 for full details.

### 2.3 Outcomes and variables of interest

The primary outcome measures were access to primary care and a previous negative HIV test. Primary care represents integration into healthcare services, beyond attendance at HIV clinics. Access to primary care was defined as possession of a health card (Italy/Spain), regular follow up with the Infectious Diseases Unit (Greece) or registration with a general practitioner (GP) or family doctor (all other countries) at the time of survey completion. Participants were asked if they had ever had a negative HIV test (year and country) and where possible missing self‐reported data were replaced by data from clinical records. Previous HIV testing was used as a marker of access to HIV prevention opportunities (e.g. how well messages promoting frequent HIV testing are reaching migrants before diagnosis) and analysis of this variable was restricted to those diagnosed post‐migration.

Data are presented by three gender‐related groups (women, heterosexual men and gay/bisexual men) as it was assumed that the three groups were all likely to be different with regard to HIV testing history and sexual behaviour. Individuals who identified as transgender were assumed to form a distinct group and were subsequently excluded from analysis due to low numbers.

Participants were grouped according to region of birth based on United Nations Statistics Division geographic regions and sub‐regions classifications 17.

Individuals were classified as diagnosed “late” if they were diagnosed with a CD4 cell count <350 cells/mm^3^ (<200 cells/mm^3^ for “very late”) and without serological evidence (e.g. avidity testing) of recent seroconversion.

### 2.4 Statistical analysis

We undertook statistical analysis using Stata (version 14.1). We accounted for the clustering of participants at clinic and the country level by declaring countries to be strata and clinics to be primary sampling units using the complex survey functions. In descriptive analysis proportions were compared using a design‐based chi‐square equivalent test and linear regression used to compare means.

Associations between the primary outcomes and socio‐demographic/behavioural factors were analysed using logistic regression, with a random effect for clinics. Initial analysis showed that access to primary care was unexpectedly very high in some countries for some gender‐related groups (e.g. 100% of women in Italy and The Netherlands). Consequently, for each group, associations are only explored in countries where less than 95% of respondents reported access to primary care.

Factors were first analysed individually (see tables in the results section for variables included in univariate analysis) and those factors found to have significant associations with the primary outcomes (α=0.05) were incorporated into a regression model using backwards selection from a hierarchy of groups. That is, covariates were arranged into logical groupings (e.g. socioeconomic, sexual behaviour etcetera) with factors considered least important tested for possible removal first. Covariate groupings not significant at the 5% level were discarded (see Table 1). In all models, *a priori* factors (country of residence, age, region of birth, years since migration, immigration status) were included. Sensitivity analyses were conducted (1) including years since HIV diagnosis as a predefined factor in the models and (2) excluding respondents who had migrated from another country in Europe (if that was not the country of birth). Associations are reported as odds ratios (OR) and adjusted OR (aOR) with 95% confidence intervals. Tests for interaction were performed.

**Table 1:** Covariate groupings of factors significant in univariate analysis for each primary outcome, tested in multivariate analysis in decreasing order of importance (1 = most important)

Previous negative testing for HIV	Access to primary care
Women	
1. Country of residence, Age, Region of birth, Years in current country of residence, Immigration status^a^	1. Country of residence, Age, Region of birth, Years in current country of residence, Immigration status^a^
2. Antenatal service attendance in the 2 years prior to diagnosis*children	2. Employment
3. Number of lifetime sexual partners & Diagnosed with STI before HIV diagnosis	3. Any health service attendance in two years before diagnosis
4. Education level	
Heterosexual men	
1. Country of residence, Age, Region of birth, Years in current country of residence, Immigration status^a^	1. Country of residence, Age, Region of birth, Years in current country of residence, Immigration status^a^
2. Number of children cared for in the home	2. Employment
	3. Experience of hunger in past 4 weeks
Gay/Bisexual men	
1. Country of residence, Age, Region of birth, Years in current country of residence, Immigration status^a^	1. Country of residence, Age, Region of birth, Years in current country of residence, Immigration status^a^
2. Number of sexual partners in current country of residence	2. Currently on ART
3. Diagnosed with an STI before HIV diagnosis	3. Any health service attendance in 2 years before diagnosis
4. Sexual orientation	4. Employment & Income
5. Employment & Income	

a Preselected covariates included in all models. Groupings and order of importance based on a priori assumptions informed by expert insight.

## 3 Results

Of 3794 patients registered on enrolment logs, 3251 eligible HIV‐positive migrants were invited to participate and 2209 (68%) accepted and completed the survey. Participation was higher in men (75%) than in women (64%, *p *<* *0.001), and decreased with age (83% in people aged 18 to 24 years and 62% in those aged over 64 years, *p *=* *0.04). Those born in Oceania and North America (Rest of World) were most likely to participate compared with those born in Africa or Europe (91.3% vs. 62.4% vs. 64.4%, respectively, *p *<* *0.001).

In total, 2117 respondents (658 women, 1435 men and 24 transgender) with matching clinical records were available for analysis. The 24 transgender participants were excluded from analysis leaving a final sample of 2093 subjects. Respondents were from 152 different countries: 35.1% Africa; 31.6% Latin America & Caribbean and 23.0% Europe (Table 2. See Figures S1 and S2 in Additional file 2 for full data). A large proportion of the sample were men (1435/2093; 68.6%) of which 68.9% (989/1435) were men who described their sexual orientation as gay or bisexual; there were differences between the three gender‐related groups in nearly all demographic characteristics (Table 2). The majority of women and heterosexual men were born in Africa compared with gay/bisexual men (63.1% vs. 57.0% vs. 7.4%, *p *<* *0.001) who were more likely to have been born in Latin America/Caribbean (18.2% vs. 16.1% vs. 46.1%, *p *<* *0.001). Median times in Current Country of Residence (CCOR) were 7, 10 and 9 years for women, heterosexual men and gay men respectively. Other notable socio‐demographic differences were in education level, employment status, income and immigration status with gay/bisexual men more likely to report paid work, higher earnings, higher levels of education and more than three‐quarters (78.8%) reporting permanent residency compared with 51.5% of women and 58.4% of heterosexual men (Table 2).

**Table 2:** Socio‐demographic characteristics of survey respondents, by gender (men separated by sexual orientation)

	Women	Heterosexual men	Gay/bisexual men	*p* value
Total number of respondents n (row %)	658 (31.4)	446 (21.3)	989 (47.3)	
Median age in years (IQR)^a^	37 (30.9 to 44.6)	41 (34.3 to 48.4)	35 (29.4 to 41.6)	
Region of birth^a^				<0.001
Africa	415 (63.1)	254 (57.0)	73 (7.4)	
Latin America/Caribbean	120 (18.2)	72 (16.1)	456 (46.1)	
Rest of World	32 (4.9)	39 (8.7)	146 (14.8)	
Europe	91 (13.8)	81 (18.2)	314 (31.7)	
Mean age in years at migration (SD)	29.3 (9.9)	30.1 (10.0)	26.3 (8.7)	<0.001
Median years in CCOR (IQR)^a^	7 (4.1 to 12.7)	10 (6.1 to 15.0)	9 (4.8 to 13.9)	
Ethnicity (n = 1881)^b^				<0.001
Black African/Caribbean	334 (59.5)	205 (51.8)	51 (5.5)	
White European	92 (16.4)	69 (17.4)	296 (32.0)	
Latin American/Hispanic	39 (7.0)	26 (6.6)	177 (19.2)	
Mixed Ethnicity	44 (7.8)	30 (7.6)	204 (22.1)	
Other	52 (9.3)	66 (16.7)	196 (21.2)	
Education: upper secondary or more^a^	322 (48.9)	228 (51.1)	802 (81.1)	<0.001
Employment status: working full/part time^a^	276 (41.9)	217 (48.7)	666 (67.3)	<0.001
Relationship status^a^				0.005
Married/Cohabitating	273 (41.5)	195 (43.7)	352 (35.6)
Single	302 (45.9)	170 (38.1)	513 (51.9)
Living apart relationship/marriage	83 (12.6)	81 (18.2)	124 (12.5)
Has children^a^	474 (72.6)	301 (69.2)	97 (9.9)	<0.001
Religion of those who attend services (n = 1165)^a^				
Christian (All denominations)	428 (85.8)	235 (76.1)	306 (85.7)	<0.001
Muslim	48 (9.6)	67 (21.7)	13 (3.6)	
Other	23 (4.6)	7 (2.3)	38 (10.6)	
Sexual orientation (n = 2076)^a^				<0.001
Gay/Lesbian	12 (1.8)	0 (0.0)	843 (85.2)	
Heterosexual	616 (94.5)	417 (95.9)	0 (0.0)	
Bisexual	14 (2.1)	0 (0.0)	146 (14.8)	
Other	10 (1.5)	18 (4.1)	0 (0.0)	
Monthly income compared to national minimum wage (n = 1975)^a^				<0.001
More or a lot more	65 (10.6)	60 (14.3)	430 (45.6)	
About the same	82 (13.4)	70 (16.7)	167 (17.7)	
Less than minimum wage	215 (35.0)	126 (30.1)	189 (20.1)	
Own wage not earned	236 (38.4)	148 (35.3)	140 (14.7)	
Not known	16 (2.6)	15 (3.6)	16 (1.7)	
Moderate/severe household hunger in past 4 weeks (n = 2006)^a^	136 (21.8)	112 (26.8)	124 (12.8)	<0.001
Immigration status (n = 2078)^a^				<0.001
Permanent residency permit	335 (51.5)	258 (58.4)	777 (78.8)	
Temporary residency permit	238 (36.6)	147 (33.3)	152 (15.4)	
Asylum seeker/Refugee status	77 (11.8)	37 (8.4)	57 (5.8)	
Unknown	58 (8.9)	23 (2.2)	48 (4.9)	
Travelled back to country of birth in past year	191 (29.0)	133 (29.8)	497 (50.3)	<0.001

Data are n (%), median (Inter‐quartile range) or mean (Standard Deviation). N = 2093 unless otherwise stated.

a Tested as an independent predictor in univariate analysis for both outcomes.

b Excludes Portugal due to restrictions on data collection. CCOR=Current Country of Residence.

### 3.1 Access to testing and care pre‐diagnosis

Table 3 shows HIV testing history and clinical characteristics of respondents at the time of diagnosis. Late (or very late) HIV diagnosis was a feature in all groups (50.0% of women, 56.9% of heterosexual men and 29.8% of gay/bisexual men were diagnosed late); most respondents were diagnosed post‐migration, with median times to diagnosis five, eight and seven years for women, heterosexual men and gay men respectively (Table 3). Health service attendance in the two years prior to diagnosis was high (>70%) in all groups but less than a quarter of women, 26% of heterosexual men and 38.9% of gay/bisexual men recalled HIV testing being mentioned/discussed at that time. Of those who had visited a GP before being diagnosed, only 11.4% of women, 21.0% of men and 28.6% of gay/bisexual men recalled being offered an HIV test. Recollections of provider‐initiated HIV test discussions in sexual health clinics were higher: 66% of women, 69.6% of heterosexual men and 73.2% of gay/bisexual men. Less than half of women recalled being offered a test in antenatal care (Table 3).

**Table 3:** Characteristics of survey respondents by gender (men separated by sexual orientation) at time of diagnosis

	Women	Heterosexual men	Gay/bisexual men
Median age in years at diagnosis (IQR)	34 (28.4 to 41.8)	38 (31.7 to 45.7)	34 (28.4 to 41.8)
Median CD4 cell count at diagnosis (IQR) (n = 15)^a^	277 (124 to 438)	240 (85 to 409)	450 (276 to 639)
Late diagnosis (n = 1815)^a^^,^ b ^,^ c			
Diagnosed <350 cells mm^3^	293 (50.0)	227 (56.9)	248 (29.8)
Diagnosed <200 cells mm^3^	173 (29.5)	148 (37.1)	110 (13.3)
Median years between migration to CCOR and diagnosis (n = 1859)^a^	5 (1 to 10)	8 (3 to 13)	7 (3 to 12)
Diagnosed in CCOR (n = 2081)^c^	598 (91.7)	416 (93.9)	864 (87.6)
AIDS defining illness within 3 months of diagnosis (n = 1997)	101 (16.0)	86 (20.5)	63 (6.7)
<1 year between negative test and diagnosis (n = 1315)	21 (6.8)	18 (9.5)	181 (22.2)
Attended health services in the 2 years prior to diagnosis (n = 1878)^a^^,^ c	423 (70.7)	310 (74.5)	717 (83.0)
Can recall mention of HIV testing at health service before diagnosis (n = 1448)^a^^,^ c	105 (24.8)	81 (26.3)	279 (38.9)
Place where offered HIV test before diagnosis^a^			
Antenatal (n = 55)	26 (49)	–	–
Inpatient (n = 255)	24 (29.6)	13 (22.4)	24 (27.9)
Emergency (n = 322)	5 (5.7)	5 (6.9)	13 (8.0)
Sexual health clinic or HIV testing clinic (n = 257)	14 (66.7)	16 (69.6)	156 (73.2)
Outpatient (n = 317)	15 (15.2)	15 (23.8)	35 (22.6)
GP/family doctor (n = 690)	23 (11.4)	32 (21.3)	97 (28.6)
Other services (n = 143)	14 (22.6)	9 (33.3)	13 (24.1)
Place of diagnosis (n = 1878)^a^^,^ c			
Antenatal service	74 (12.4)	3 (0.7)	3 (0.3)
Hospital service, e.g. Emergency/Inpatient/Outpatient	240 (40.1)	196 (47.2)	171 (19.8)
Sexual health clinic or HIV testing clinic	75 (12.5)	66 (15.9)	376 (43.5)
GP/Family Doctor	105 (17.5)	95 (22.9)	201 (23.3)
Private clinic	17 (2.8)	7 (1.7)	43 (5.0)
Other	88 (14.7)	48 (11.6)	70 (8.1)
Tested because unwell/health problems^c^	261 (39.7)	230 (51.6)	256 (25.9)
Tested because of perceived risk^c^	128 (19.5)	77 (17.3)	384 (38.8)
Previous self‐reported negative HIV test (n = 2028)^d^	294 (46.7)	183 (43.4)	801 (82.0)
Country of previous negative test (n = 1258)			
Current country of residence	128 (44.3)	95 (54.0)	524 (66.1)
Country of birth	145 (50.2)	67 (38.1)	218 (27.5)
Other country	16 (5.5)	14 (8.0)	51 (6.4)

Data are n (%), median (Inter‐quartile range) or mean (Standard Deviation). CCOR=Current Country of Residence. N = 2093 unless otherwise stated.

a Individuals diagnosed in current country of residence only.

b Individuals diagnosed with serological evidence of seroconversion (e.g. avidity testing) excluded.

c Tested as an independent predictor in univariate analysis.

d Data missing from self‐report supplemented from clinic records.

There were high rates (82.8%) of previous negative testing among migrant gay/bisexual men, but less than half of women and heterosexual men (46.9% and 43.9%, respectively) reported ever having had a negative test (Table 3).

Among women, those who received antenatal care in CCOR (post‐migration) were three times as likely to have had a previous negative test (aOR 3.21 95% CI 1.55 to 6.66) than parous women who had not received antenatal care post‐migration (Table 4). Multivariable analysis among heterosexual men found previous negative testing was significantly associated with immigration status, with those with temporary residency significantly less likely to have had a previous test than those with permanent residency (aOR 0.41 95% CI 0.23 to 0.75; Table 4) after adjusting for CCOR, age, region of birth and years since migration. Among gay/bisexual men, negative testing was significantly associated with: CCOR, age, total number of sexual partners in CCOR, previous diagnosis with a sexually transmitted infection and sexual orientation—with bisexual men being less likely (aOR 0.43 95% CI 0.25 0.74) to have had a previous negative test than gay men (Table 4). Sensitivity analyses did not indicate that including the number of years since HIV diagnosis would improve the multivariable models and did not affect the associations of the other factors. Sensitivity analysis excluding respondents who had migrated from another country in Europe (n = 188) did not appreciably alter the findings.

**Table 4:** Factors associated with self‐reported previous HIV‐negative test^a^, among women, heterosexual men and gay or bisexual men living with diagnosed HIV post‐migration and attending HIV clinics in Europe

	% (n/N)	OR	AOR	95% CI	*p* value
Women (N = 565)					
Current country of residence (CCOR)					0.233
Belgium	53.8 (50/93)	1.59	1.49	0.72 to 3.10	
Greece	29.5 (18/61)	0.57	0.68	0.30 to 1.53	
Germany	66.7 (6/9)	3.19	3.21	0.68 to 15.18	
Italy	26.1 (6/23)	0.48	0.59	0.19 to 1.86	
Netherlands	50.0 (10/20)	1.37	1.79	0.61 to 5.27	
Portugal	50.0 (38/76)	1.40	1.49	0.74 to 3.02	
Spain	42.3 (60/142)	1.00	1.00	–	
Switzerland	44.9 (22/49)	1.04	1.41	0.63 to 3.20	
United Kingdom	58.7 (54/92)	1.96	1.76	0.90 to 3.45	
Age					0.154
18 to 24	35.1 (13/37)	0.57	0.71	0.32 to 1.57	
25 to 34	49.5 (100/202)	1.00	1.00	–	
35 to 44	48.4 (90/186)	0.96	0.85	0.54 to 1.33	
45 to 54	48.5 (48/99)	0.93	0.81	0.47 to 1.41	
55+	31.7 (13/41)	0.47	0.36	0.16 to 0.80	
Region of birth					0.305
Africa	50.6 (176/348)	1.00	1.00	–	
Latin America/Caribbean	44.5 (49/110)	0.78	0.79	0.46 to 1.36	
Rest of World	28.0 (7/25)	0.38	0.42	0.15 to 1.17	
Europe	39.0 (32/82)	0.62	0.73	0.41 to 1.30	
Years resident in country					0.050
≤2	41.9 (18/43)	0.70	0.57	0.26 to 1.25	
3 to 5	38.0 (57/150)	0.56	0.48	0.28 to 0.82	
6 to 10	49.1 (80/163)	0.92	0.82	0.52 to 1.31	
>10	52.2 (109/209)	1.00	1.00	–	
Immigration status					0.327
Permanent residency	50.7 (151/298)	1.00	1.00	–	
Temporary residency	41.7 (70/168)	0.69	0.72	0.46 to 1.13	
Refugee/Asylum seeker/Unknown	43.4 (43/99)	0.76	0.77	0.44 to 1.32	
Has children					0.006
No children	44.7 (67/150)	1.04	1.00	0.64 to 1.54	
Has children, no antenatal care in CCOR	43.9 (161/367)	1.00	1.00	–	
Has children, received antenatal care in CCOR	75.0 (36/48)	3.88	3.21	1.55 to 6.66	
Heterosexual men (N = 379)					
Current country of residence (CCOR)					0.106
Belgium	57.1 (28/49)	1.78	2.51	1.14 to 5.52	
Greece	32.0 (16/50)	0.63	0.68	0.30 to 1.55	
Germany	25.0 (2/8)	0.44	0.43	0.08 to 2.42	
Italy^b^	0.0 (0/15)	–	–	–	
Netherlands	52.6 (10/19)	1.48	2.24	0.79 to 6.33	
Portugal	40.0 (16/40)	0.89	0.92	0.42 to 2.04	
Spain	42.9 (48/112)	1.00	1.00	–	
Switzerland	53.5 (23/43)	1.53	1.41	0.65 to 3.06	
United Kingdom	51.7 (30/58)	1.42	1.55	0.76 to 3.14	
Age (years)					0.092
18 to 24	30.0 (3/10)	0.39	0.36	0.08 to 1.56	
25 to 34	50.5 (47/93)	0.92	0.88	0.50 to 1.56	
35 to 44	50.7 (73/144)	1.00	1.00	–	
45 to 54	39.4 (37/94)	0.65	0.58	0.33 to 1.00	
55+	34.2 (13/38)	0.54	0.42	0.19 to 0.94	
Region of birth					0.578
Africa	44.2 (91/206)	1.00	1.00	–	
Latin America/Caribbean	48.4 (31/64)	1.26	1.32	0.69 to 2.52	
Rest of World	38.2 (13/34)	0.82	1.10	0.48 to 2.54	
Europe	50.7 (38/75)	1.35	1.55	0.81 to 2.97	
Years resident in country					0.428
≤2	50.0 (5/10)	0.98	1.96	0.47 to 8.22	
3 to 5	45.5 (25/55)	0.90	1.25	0.60 to 2.60	
6 to 10	51.4 (56/109)	1.34	1.54	0.90 to 2.64	
>10	42.4 (87/205)	1.00	1.00	–	
Immigration status					0.013
Permanent residency	50.0 (117/234)	1.00	1.00	–	
Temporary residency	33.7 (29/86)	0.42	0.41	0.23 to 0.75	
Refugee/Asylum seeker/Unknown	45.8 (27/59)	0.80	0.55	0.27 to 1.15	
Gay or bisexual men (n = 780)					
Current country of residence					<0.001
Belgium	83.1 (49/59)	1.13	0.65	0.27 to 1.60	
Greece	44.4 (20/45)	0.15	0.12	0.05 to 0.29	
Germany	50.0 (3/6)	0.26	0.17	0.02 to 1.28	
Italy^b^	0.0 (0/4)	1.00	1.00	–	
Netherlands	90.3 (56/62)	1.75	1.33	0.48 to 3.69	
Portugal	91.1 (41/45)	1.72	3.41	1.11 to 10.50	
Spain	83.8 (299/357)	1.00	1.00	–	
Switzerland	78.4 (29/37)	0.66	0.63	0.24 to 1.67	
United Kingdom	89.3 (151/169)	1.71	1.06	0.53 to 2.13	
Age (years)					0.045
18 to 24	70.6 (48/68)	0.54	0.43	0.22 to 0.85	
25 to 34	83.8 (269/321)	1.00	1.00	–	
35 to 44	83.6 (219/262)	0.99	0.90	0.54 to 1.51	
45 to 54	90.3 (93/103)	1.61	1.87	0.82 to 4.25	
55+	73.1 (19/26)	0.57	1.10	0.33 to 3.62	
Region of birth					0.292
Africa	76.9 (40/52)	0.67	1.36	0.54 to 3.40	
Latin America/Caribbean	83.3 (319/383)	1.00	1.00	–	
Rest of World	81.3 (87/107)	0.84	1.17	0.55 to 2.51	
Europe	84.9 (202/238)	1.02	1.84	0.97 to 3.50	
Years resident in country					0.128
≤2	90.7 (39/43)	1.65	3.71	1.09 to 12.66	
3 to 5	80.3 (118/147)	0.87	1.79	0.90 to 3.53	
6 to 10	82.8 (212/256)	0.98	1.13	0.67 to 1.91	
>10	83.5 (279/334)	1.00	1.00	–	
Immigration status					0.125
Permanent residency	85.4 (527/617)	1.00	1.00	–	
Temporary residency	78.1 (89/114)	0.63	0.72	0.38 to 1.37	
Refugee/Asylum seeker/Unknown	65.3 (32/49)	0.35	0.44	0.20 to 0.99	
Number of sexual partners in current country of residence					0.008
0 to 5	81.3 (109/134)	0.33	0.41	0.18 to 0.93	
6 to 10	72.6 (61/84)	0.18	0.32	0.14 to 0.74	
11 to 20	81.4 (79/97)	0.33	0.41	0.18 to 0.93	
21 to 50	73.4 (94/128)	0.21	0.24	0.12 to 0.49	
51 to 100	86.0 (104/121)	0.46	0.46	0.21 to 1.02	
More than 100	93.1 (201/216)	1.00	1.00	–	
Diagnosed with STI before HIV diagnosis					<0.001
No	75.2 (354/471)	1.00	1.00	–	
Yes	95.1 (294/309)	6.42	4.41	2.42 to 8.03	
Sexual orientation					0.002
Gay	85.4 (568/665)	1.00	1.00	–	
Bisexual	69.6 (80/115)	0.37	0.43	0.25 to 0.74	

OR, odds ratio; AOR, adjusted odds ratio; 95% CI, 95% confidence interval; ART, antiretroviral therapy; STI, sexually transmitted infection.

a After final model selection. All models adjusted for factors listed in the model.

b Excluded from multivariable analysis because of perfect prediction (separation).

### 3.2 Access to treatment and ongoing care

Most participants in all groups were on antiretroviral treatment and 77.2% of women, 75.9% of heterosexual men and 77.9% of gay/bisexual men on treatment had an undetectable viral load (<50 copies/mL; Table 5). Most of those not on treatment reported this was because of their doctor's advice or because they were newly diagnosed. Around a third of women (32.2%) had experienced difficulties with health services since migration, a third of whom cited long waiting times in clinics, 22% did not trust GP confidentiality while 19.9% said they were unclear of their legal rights to access care (Table 5). Slightly fewer men of either sexual orientation group reported difficulties overall. Among gay/bisexual men, long waiting times were a problem for 40.1% who reported difficulties, whereas 25.3% were unclear of their rights to access care. For heterosexual men who reported problems, language barriers presented difficulties for 27.7% and a quarter (25.3%) were unclear of their rights to access care.

**Table 5:** HIV treatment characteristics of aMASE clinic survey respondents, by gender (men separated by sexual orientation)

** **	Women	Heterosexual men	Gay/bisexual men
Most recent CD4 cell count ≥350 cells mm3 (n = 2011)	494 (76.8)	282 (65.4)	814 (86.9)
Undetectable viral load (<50 copies/mL) (n = 1540)^a^	409 (77.2)	290 (75.9)	489 (77.9)
Currently not on HIV treatment (n = 2090)^b^	105 (16.0)	40 (9.0)	312 (31.6)
Reason not on HIV treatment (n = 457)			
Doctor's advice or newly diagnosed	90 (85.7)	33 (82.5)	276 (88.5)
High cost or otherwise inaccessible	3 (2.9)	0 (0.0)	15 (4.8)
Fear of side effects or other difficulties taking medication	9 (8.6)	5 (12.5)	25 (8.0)
Other reason	7 (6.7)	3 (7.5)	16 (5.1)
Access to primary care (n = 2076)	552 (85.1)	369 (83.5)	833 (84.6)
Government‐funded HIV treatment and care (n = 972)^b^^,^ c	244 (78.2)	162 (78.6)	319 (70.3)
Experienced difficulties with health service in CCOR (n = 2093)	211 (32.3)	132 (29.9)	272 (27.7)
No GP/Health card/insurance (n = 628)	33 (15.3)	18 (13.1)	58 (20.9)
Unclear of rights to access medical care (n = 629)	43 (19.9)	35 (25.5)	70 (25.3)
Long waiting times for an appointment/in the clinic (n = 628)	72 (33.3)	29 (21.2)	111 (40.1)
Does not trust the GP confidentiality (n = 628)	48 (22.2)	31 (22.6)	37 (13.4)
Difficulty communicating with staff because of language differences (n = 628)	55 (25.5)	38 (27.7)	38 (13.7)
Difficulty negotiating healthcare system (e.g. finding GP, payment, travel) (n = 629)	22 (10.2)	13 (9.5)	31 (11.2)
Missed clinical appointments because of travel expenses (n = 2071)	77 (11.9)	66 (15.1)	68 (6.9)
Delayed/forwent medication because of prescription costs (n = 2078)^d^	54 (8.3)	39 (8.8)	48 (4.9)

Data are n (%).CCOR, Current Country of Residence.

a Only those on antiretroviral therapy (ART).

b Tested as an independent predictor in univariate analysis.

c Excludes co‐pays.

d Includes medicines other than antiretroviral therapy.

Travel expenses and prescription costs presented additional barriers for those who funded this element of their care. The cost of prescriptions (for all medication, not just Antiretrovirals) resulted in delaying or forgoing medications for 8.3% of women, 8.8% of heterosexual men and 4.9% of gay/bisexual men. Around one in ten women (11.9%), 15.1% of heterosexual men and 6.9% of gay/bisexual men reported missing appointments due to travel costs (Table 5).

Access to primary care was varied across countries. In Greece, Germany, Italy, the Netherlands and United Kingdom >95% of respondents in one or more gender‐related group reported having a primary care physician or access to an infectious disease unit (Table 6). In multivariable analysis, access to primary care was associated with CCOR and immigration status in all three groups. In addition, years since migration and being on antiretroviral therapy remained significantly associated with access to primary care among gay/bisexual men.

**Table 6:** Factors associated with access to primary care^a^ among women, heterosexual men and gay/bisexual men living with diagnosed HIV and attending HIV clinics in Europe

	% (n/N)	OR	AOR	95% CI	*p* value
Women (N = 409)					
Current country of residence					<0.001
Belgium	81.5 (88/108)	0.38	0.42	0.17 to 1.04	
Greece^b^	98.5 (65/66)	–	–	–	
Germany^b^	80.0 (8/10)	–	–	–	
Italy^b^	100.0 (35/35)	–	–	–	
Netherlands^b^	100.0 (20/20)	–	–	–	
Portugal	54.8 (46/84)	0.10	0.09	0.04 to 0.21	
Spain	92.1 (140/152)	1.00	1.00	–	
Switzerland	66.2 (43/65)	0.17	0.22	0.09 to 0.56	
United Kingdom^b^	98.2 (107/109)	–	–	–	
Age (years)					0.129
18 to 24	71.4 (20/28)	0.69	0.47	0.17 to 1.28	
25 to 34	78.4 (120/153)	1.00	1.00	–	
35 to 44	80.0 (108/135)	1.10	1.04	0.54 to 1.98	
45 to 54	81.5 (53/65)	1.21	1.24	0.52 to 2.95	
55+	57.1 (16/28)	0.37	0.39	0.14 to 1.08	
Region of birth					0.420
Africa	74.5 (190/255)	1.00	1.00	–	
Latin America/Caribbean	86.9 (86/99)	2.26	0.90	0.39 to 2.06	
Rest of World	61.5 (8/13)	0.55	0.35	0.09 to 1.34	
Europe	78.6 (33/42)	1.25	0.66	0.25 to 1.70	
Years resident in country					0.252
≤2	62.8 (27/43)	0.26	0.40	0.14 to 1.12	
3 to 5	71.7 (91/127)	0.39	0.45	0.20 to 1.03	
6 to 10	79.3 (88/111)	0.59	0.60	0.27 to 1.35	
>10	86.7 (111/128)	1.00	1.00	–	
Immigration status					0.028
Permanent residency	84.0 (178/212)	1.00	1.00	–	
Temporary residency	68.1 (81/119)	0.41	0.41	0.22 to 0.79	
Refugee/Asylum seeker/Unknown	74.4 (58/78)	0.55	0.56	0.26 to 1.21	
Heterosexual men (N = 271)					
Current country of residence					0.004
Belgium	63.3 (38/60)	0.25	0.34	0.14 to 0.85	
Greece^b^	96.3 (52/54)	–	–	–	
Germany^b^	100.0 (9/9)	–	–	–	
Italy^b^	100.0 (23/23)	–	–	–	
Netherlands^b^	90.5 (19/21)	–	–	–	
Portugal	72.1 (31/43)	0.37	0.24	0.09 to 0.61	
Spain	87.4 (104/119)	1.00	1.00	–	
Switzerland	65.3 (32/49)	0.27	0.22	0.09 to 0.56	
United Kingdom^b^	95.3 (61/64)	–	–	–	
Age (years)					0.211
18 to 24	62.5 (5/8)	0.56	0.44	0.08 to 2.40	
25 to 34	80.0 (56/70)	1.33	1.40	0.61 to 3.21	
35 to 44	75.0 (75/100)	1.00	1.00		
45 to 54	69.7 (46/66)	0.77	0.56	0.26 to 1.22	
55+	85.2 (23/27)	1.92	1.72	0.49 to 6.04	
Region of birth					0.572
Africa	68.9 (104/151)	1.00	1.00		
Latin America/Caribbean	83.3 (50/60)	2.26	1.27	0.52 to 3.09	
Rest of World	71.4 (10/14)	1.13	1.07	0.28 to 4.00	
Europe	89.1 (41/46)	3.71	2.18	0.72 to 6.57	
Years resident in country					0.879
≤2	64.3 (9/14)	0.38	1.11	0.28 to 4.45	
3 to 5	57.4 (27/47)	0.29	0.72	0.28 to 1.88	
6 to 10	77.4 (65/84)	0.72	0.87	0.39 to 1.95	
>10	82.5 (104/126)	1.00	1.00		
Immigration status					0.040
Permanent residency	84.9 (135/159)	1.00	1.00	..	
Temporary residency	67.7 (44/65)	0.26	0.40	0.18 to 0.88	
Refugee/Asylum seeker/Unknown	55.3 (26/47)	0.22	0.34	0.13 to 0.92	
Gay/bisexual men (N = 913)					
Current country of residence					<0.001
Belgium	75.6 (65/86)	0.40	0.31	0.11 to 0.86	
Greece^b^	96.4 (53/55)	–	–	–	
Germany^b^	100.0 (12/12)	–	–	–	
Italy^b^	100.0 (5/5)	–	–	–	
Netherlands	94.9 (74/78)	2.38	1.62	0.43 to 6.12	
Portugal	52.9 (27/51)	0.14	0.06	0.02 to 0.16	
Spain	88.6 (365/412)	1.00	1.00	–	
Switzerland	59.0 (36/61)	0.19	0.08	0.03 to 0.21	
United Kingdom	87.1 (196/225)	0.87	0.55	0.24 to 1.26	
Age					0.334
18 to 24	72.6 (61/84)	0.66	0.69	0.36 to 1.30	
25 to 34	80.0 (308/385)	1.00	1.00	–	
35 to 44	86.0 (257/299)	1.53	1.03	0.62 to 1.70	
45 to 54	94.3 (115/122)	4.11	2.11	0.85 to 5.26	
55+	95.7 (22/23)	5.50	1.34	0.16 to 11.34	
Region of birth					0.255
Africa	83.3 (55/66)	1.06	2.36	0.95 to 5.88	
Latin America/Caribbean	82.4 (371/450)	1.00	1.00	–	
Rest of World	85.7 (114/133)	1.28	1.49	0.74 to 3.00	
Europe	84.5 (223/264)	1.16	1.13	0.63 to 2.01	
Years resident in country					<0.001
≤2	59.8 (58/97)	0.15	0.17	0.09 to 0.35	
3 to 5	72.6 (127/175)	0.27	0.44	0.24 to 0.82	
6 to 10	89.6 (250/279)	0.89	1.21	0.66 to 2.22	
>10	90.6 (328/362)	1.00	1.00	–	
Immigration status					<0.001
Permanent residency	88.0 (639/726)	1.00	1.00	–	
Temporary residency	70.2 (92/131)	0.33	0.48	0.28 to 0.85	
Refugee/Asylum seeker/Unknown	57.1 (32/56)	0.15	0.21	0.10 to 0.45	
Currently on ART					0.001
No	79.9 (231/289)	0.69	0.42	0.26 to 0.70	
Yes	85.3 (532/624)	1.00	1.00	–	

OR, odds ratio; AOR, adjusted odds ratio; 95% CI, 95% confidence interval; ART, antiretroviral therapy.

a After final model selection.

b Excluded from multivariable analysis because of perfect prediction (separation) or small numbers. All models adjusted for factors listed in the table.

There were no significant interactions between CCOR or country of birth and any of the factors in the models selected for any group (data not shown).

## 4 Discussion

This study provides valuable data about the barriers and facilitators to secondary HIV prevention and accessing primary care for different migrant groups living with HIV in Europe. In addition, we have shown that for migrant women and heterosexual men, structural factors related to child‐bearing or immigration status have a strong association with access to HIV testing. For migrant gay/bisexual men barriers to testing are mainly related to sexual behavioural factors with bisexual men and those with fewer partners less likely to have a previous negative test. Access to primary care, an indicator of integration into health services, was found to be strongly associated with current country of residence in all groups and immigration status among women and gay/bisexual men.

### 4.1 Policy Implications

Our findings suggest that for migrant women and heterosexual men, interventions that target sexual behaviour or other individual‐level lifestyle factors might not be particularly successful in increasing the uptake of HIV testing. Rather, interventions that aim to address structural barriers could achieve more in the effort to increase access to earlier and regular testing. Large numbers in each group had attended primary care in the two years prior to diagnosis, however, attendance was not associated with the probability of having a negative test before diagnosis, or was it associated with late diagnosis (data not shown). The low proportion of individuals offered an HIV test before diagnosis suggests that there are continued missed opportunities for HIV testing, particularly in primary care. Policies advocating opportunistic provider‐initiated testing, as provided in antenatal services in much of Europe, have been successful in increasing HIV testing and diagnosing women at earlier stages of infection 18 and a similar approach could work in primary care. Previous studies have shown that the introduction of routine, rapid or point‐of‐care testing in primary care is feasible and acceptable, especially among migrant or black and minority ethnic communities 19, 20, 21, 22. In addition, there may still be missed opportunities for testing in antenatal care as less than half of those who attended recalled HIV being mentioned during their visit. It is possible that women were unaware of routine opt‐out testing. However, as multivariable analysis indicated that previous negative testing was associated with post‐migration antenatal care these findings are difficult to interpret.

Missed opportunities are also likely to shape the HIV epidemic among migrant gay and bisexual men. Findings from this study support others that suggest that health promotion specialists may wish to consider targeted HIV testing interventions with men who identify as bisexual or who have low numbers of sexual partners 23, 24, 25, 26. Although the vast majority of gay/bisexual men had previously tested negative for HIV, over half had seroconverted within two years of their last negative test. This suggests there remain unmet HIV prevention needs, particularly about safer sex and condom use in this group, as highlighted by other studies 23, 24, 25, 26, 27. Policymakers might need to expedite access to biomedical interventions such as Pre‐exposure Prophylaxis (PrEP) that have been shown to be highly effective in European contexts 28, 29 for migrant MSM and focus combination prevention efforts on this group in an effort to reduce seroconversion. Incorporating migration status with transmission risk in national surveillance data will enhance the ability to monitor and address HIV prevention needs for migrant MSM. In this study, we have shown that the main barriers to accessing ongoing healthcare are similar to those expressed by patients living with other chronic conditions, for example, long waiting times and difficulties with appointments 30, 31. This is perhaps to be expected as the mechanisms of accessing care are well documented as barriers, particularly among migrants who have competing interests which deprioritize health concerns 10.

Immigration legislation differs across Europe and changes to health policy affecting migrants frequently occur 32, 33. While all countries in this survey currently provide free ART for at least some migrants, only the United Kingdom provides all migrants with free ART regardless of their immigration status (see OptTest for more details 33). However, even in the United Kingdom such affordable healthcare does not extend to other health conditions, which may present challenges for migrants living with multimorbidities.

Within this survey a substantial proportion of respondents were not taking ART, possibly because the data from this study were collected before updated European HIV Treatment Guidelines recommended immediate ART initiation irrespective of CD4 count 34. The impact of the new guidelines on the uptake of ART in this population cannot be known from this study and further research is needed to establish if there remains a substantial (16 to 31%) deficit in uptake of ART, particularly among migrant gay and bisexual men, and whether high treatment costs are a barrier to ART initiation or adherence for migrants who are not entitled to free ART. To highlight the benefits of HIV treatment, clinicians and policymakers should consider the enhanced promotion of campaigns such as “Undetectable = Untransmittable” to migrants have not received accurate and up‐to‐date information about the risks of sexual transmission of HIV for those successfully on ART 35, 36.

Migration‐specific barriers, such as language barriers and difficulties understanding the legal rights to accessing healthcare, presented a problem for 20 to 25% of participants who experienced difficulties, although this finding is likely to have been underestimated (see below). These barriers may present challenges to physicians providing complex ongoing HIV healthcare. For example, language barriers could lead to poor health literacy among patients and consequently impact on the initiation of—and adherence to—ART as well as potentially facilitating onward transmission 37. While it is beyond the scope of this survey to ascertain whether uncertainty surrounding the legal rights to access care leads to poor clinic attendance or adherence, other studies have shown that fear of deportation has prevented individuals from seeking care^10^. In addition, as some healthcare providers seek to normalize HIV by shifting care aware from specialist services to general practice, this study presents a timely understanding of some of the potential barriers to such policies.

Finally, this study found that poverty may influence access to ongoing care, with a substantial proportion of all groups reporting missing clinic appointments due to travel expenses and delaying or foregoing medication due to prescription costs. Poverty was especially prevalent among heterosexual men with over 20% reporting moderate or severe household hunger in the past four weeks. Poverty is well recognized as being associated with poorer engagement in care 38, 39, 40, 41. While tackling the overall problem of economic inequality is beyond the capacity of service providers, these impediments to care need to be recognized and where possible support offered to help mitigate this barrier.

### 4.2 Limitations

This study is not without its limitations 16. The clinics and the countries were not selected at random and as such this is a convenience sample and therefore some of the prevalence estimates may have been over‐ or underestimated. In particular, it is likely that access to primary care was overestimated in some countries, as by including “health cards” and “infectious disease units” in our definition of primary care we may not have been able to sufficiently distinguish between family doctors/GPs and specialist care for HIV; therefore, caution is urged when using these estimates in health service planning. The proportions of respondents experiencing difficulties accessing health services are likely to have been underestimated, as those who experienced the greatest difficulties would not have been available in clinic to be recruited to the survey. It was assumed that those without a previous negative HIV test had experienced barriers to HIV testing up until the point of diagnosis. It is possible that some individuals had tested for the first time immediately after being exposed to HIV risk. However, given that a large proportion of participants were diagnosed late, it is likely that this previous negative testing is a suitable proxy for access to HIV testing.

## 5 Conclusion

Migrants are accessing healthcare in Europe prior to HIV diagnosis. While many migrants had previously tested negative for HIV, missed opportunities for earlier diagnosis persist among all migrant groups. In gay and bisexual migrant men many of who initially tested HIV negative in the receiving country went on to acquire HIV at a later date. Interventions to further expand testing outside of sexual health and antenatal settings are still required and these opportunities should be linked with combination prevention measures such as access to PrEP and treatment as prevention.

## Competing interests

The authors of this manuscript have no competing interests to declare.

## Authors’ contributions

JDA and FB initiated this project. All authors and contributors in acknowledgements section were involved in data collection and exchange. IF carried out the data analyses and drafted the initial manuscript. FB, JDA and AC were also involved in analysis interpretation and contributed to the discussion and conclusions. AC also provided statistical support. All authors contributed to the design of the study, commented on the manuscript and approved the final draft.

## Supporting information


**Additional File 1.** Ethical approval for the aMASE (advancing Migrant Access to Health Services in Europe) studies in each participating country.
**Additional File 2.**
**Figure S1.** Current country of residence of male and female respondents to the aMASE Clinic Survey. N = 2093 from 57 clinic sites (min, max patients 1, 148): Belgium 255, 4 clinics (27, 148; Germany 31, 2 clinics (14, 17; Greece 175, 8 clinics (1, 60) Italy 63, 2 clinics (20, 43); Netherlands 119, 3 clinics (28, 51); Portugal 179, 7 clinics (5, 54); Spain 693, 18 clinics (9, 141); Switzerland 177, 6 clinics (5, 42); United Kingdom 401, 7 clinics (21, 106).
**Figure S2.** Country of birth of male and female respondents to the aMASE clinic survey. N = 2093 from 152 countries. Brazil 146; Colombia 107; Nigeria 96; Ecuador 74; Cameroon 59; Ghana 58; Venezuela 57; Romania 56; Italy 52; Guinea‐Bissau 49; Albania 43; Peru 39; Cuba 37; Argentina 36; Dominican Republic 36; Congo (Kinshasa) 35; Portugal 35; Poland 34; Russia 33; Spain 32; France 31; Guinea 30; Angola 28; Equatorial Guinea 27; Morocco 27; Ukraine 26; Cote d'Ivoire 25; Zimbabwe 25; Cape Verde 24; United States of America 23; Bulgaria 22; Eritrea 22; Ethiopia 20; United Kingdom 20; Georgia 19; Rwanda 19; Togo 19; Bolivia 17; Mozambique 17; South Africa 17; Kenya 16; China 15; Paraguay 15; Thailand 15; Germany 14; Mexico 14; Suriname 13; Burundi 12; Philippines 12; Sierra Leone 12; Turkey 12; India 11; Chile 9; Malaysia 9; Serbia 9; Uruguay 9; Australia 8; Canada 8; Honduras 8; Hungary 8; Malawi 8; Netherlands 8; Senegal 8; Tanzania 8; Tunisia 8; Uganda 8; Benin 7; Burkina Faso 7; Czech Republic 7; Iran 7; Jamaica 7; Lebanon 7; Pakistan 7; Armenia 5; Belgium 5; Cyprus 5; Indonesia 5; Kazakhstan 5; Liberia 5; Nicaragua 5; Sweden 5; Switzerland 5; Uzbekistan 5; Finland 4; Greece 4; Hong Kong 4; Mali 4; Mauritius 4; Moldova 4; Nepal 4; Sao Tome and Principe 4; Trinidad and Tobago 4; Zambia 4; Austria 3; Congo (Brazzaville) 3; Egypt 3; Estonia 3; Gambia, The 3; Ireland 3; Israel 3; Japan 3; Latvia 3; Netherlands Antilles 3; Sri Lanka 3; Sudan 3; Vietnam 3; Afghanistan 2; Antigua and Barbuda 2; Bangladesh 2; Bosnia and Herzegovina 2; Botswana 2; Denmark 2; Gabon 2; Guatemala 2; Iraq 2; Kosovo 2; Macedonia 2; Madagascar 2; New Zealand 2; Norway 2; Panama 2; Seychelles 2; Slovakia 2; Slovenia 2; Somalia 2; Taiwan 2; Algeria 1; Azerbaijan 1; Bahamas, The 1; Barbados 1; Belarus 1; Burma 1; Central African Republic 1; Comoros 1; Croatia 1; Djibouti 1; Dominica 1; El Salvador 1; Guyana 1; Haiti 1; Laos 1; Libya 1; Lithuania 1; Mauritania 1; Niger 1; Oman 1; Swaziland 1; Syria 1; Tajikistan 1; Timor‐Leste 1; Turkmenistan 1; United Arab Emirates 1.
**Additional File 3**
**Table S2.** Sociodemographic characteristics of survey respondents, by gender (men separated by sexual orientation)
**Table S3.** Characteristics of survey respondents by gender (men separated by sexual orientation) at time of diagnosis
**Table S4.** HIV treatment characteristics of aMASE clinic survey respondents, by gender (men separated by sexual orientation)

## Appendix 1

### 1.1

This study would not be possible without the entire Work Package 14 collaboration. The aMASE study team are as follows: A Aerssens, M Aguado, B Alimi, D Álvarez‐del Arco, O Anagnostou, J Anderson, A Antoniadou, M Arando, MJ Barberà, H Barros, A Barthélemy, J Belda‐Ibáñez, B Bertisch, J Bil, JR Blanco, K Block, C Boesecke, M Boura, J Burgos, FM Burns, J Cabo, E Calabuig, L Campbell, O Cardoso, W Claudia, N Clumeck, A Colucci, S Corrao, S Cuellar, J Cunha, G Daikos, K Darling, J del Amo, J del Romero, P Dellot, M Dixneuf, P Domingo, F Dronda, F Ebeling, A Engelhardt, B Engler, I Fakoya, J Farrell, J Fehr, M Feijó, E Fernández, E Fernández García, T Fernandez, AL Fortes, J Fox, P Garcia de Olalla, F García, P Gargalianos‐Kakolyris, AF Gennotte, I Germano, G Gilleran, R Gilson, S Goepel, HA Gogos, JL Gómez Sirvent, I Gountas, A Gregg, F Gutiérrez, MM Gutierrez, I Hermans, JA Iribarren, H Knobel, L Koulai, S Kourkounti, C La Morté, T LeCompte, B Ledergerber, L Leonidou, MC Ligero, G Lindergard, S Lino, MJ Lopes, A Lopez Lirola, M Louhenapessy, G Lourida, AM Luzi, F Maltez, L Manirankunda, A Martín‐Pérez, L Martins, M Masía, MG Mateu, P Meireles, A Mendes, S Metallidis, S Mguni, A Milinkovic, JM Miró, K Mohrmann, S Monge, M Montero, T Mouhebati, M Moutschen, M Müller, C Murphy, C Nöstlinger, I Ocaña, S Okumu‐Fransche, G Onwuchekwa, JE Ospina, D Otiko, P Pacheco, R Palacios, V Paparizos, V Papastamopoulos, V Paredes, N Patel, T Pellicer, A Peña, N Petrosillo, A Pinheiro, J Poças, A Portillo, F Post, F Prestileo, T Prestileo, M Prins, P Prins, K Protopapas, M Psichogiou, F Pulido, J Rebollo, A Ribeirinho, I Río, M Robau, JK Rockstroh, E Rodrigues, M Rodríguez, C Sajani, M Salavert, R Salman, N Sanz, G Schuettfort, G Schüttfort, C Schwarze‐ Zander, R Serrão, D Silva, V Silva, P Silverio, A Skoutelis, C Staehelin, C Stephan, C Stretton, F Styles, AF Sutre, S Taylor, B Teixeira, C Thierfelder, G Touloumi, O Tsachouridou, K Tudor, E Valadas, M van Frankenhuijsen, M Vázquez, M Velasco Arribas, M Vera, P Vinciana, A Volny‐Anne, N Voudouri, JC Wasmuth, C Wengenroth, E Wilkins, L Young, S Yurdakul, T Zafra Espinosa, W Zuilhof, F Zuure.
