# Supplementary material for: HIV testing history and access to treatment among migrants living with HIV in Europe
Source: J Int AIDS Soc. 2018 Jul 19;21(Suppl Suppl 4):e25123. doi: 10.1002/jia2.25123 (PMC6053481; doi:10.1002/jia2.25123)
Supplement: Supplementary file 1 — Additional File 1. Ethical approval for the aMASE (advancing Migrant Access to Health Services in Europe) studies in each participating country. Additional File 2. Figure S1. Current country of residence of male and female respondents to the aMASE Clinic Survey. N = 2093 from 57 clinic sites (min, max patients 1, 148): Belgium 255, 4 clinics (27, 148; Germany 31, 2 clinics (14, 17; Greece 175, 8 clinics (1, 60) Italy 63, 2 clinics (20, 43); Netherlands 119, 3 clinics (28, 51); Portugal 179, 7 clinics (5, 54); Spain 693, 18 clinics (9, 141); Switzerland 177, 6 clinics (5, 42); United Kingdom 401, 7 clinics (21, 106). Figure S2. Country of birth of male and female respondents to the aMASE clinic survey. N = 2093 from 152 countries. Brazil 146; Colombia 107; Nigeria 96; Ecuador 74; Cameroon 59; Ghana 58; Venezuela 57; Romania 56; Italy 52; Guinea‐Bissau 49; Albania 43; Peru 39; Cuba 37; Argentina 36; Dominican Republic 36; Congo (Kinshasa) 35; Portugal 35; Poland 34; Russia 33; Spain 32; France 31; Guinea 30; Angola 28; Equatorial Guinea 27; Morocco 27; Ukraine 26; Cote d'Ivoire 25; Zimbabwe 25; Cape Verde 24; United States of America 23; Bulgaria 22; Eritrea 22; Ethiopia 20; United Kingdom 20; Georgia 19; Rwanda 19; Togo 19; Bolivia 17; Mozambique 17; South Africa 17; Kenya 16; China 15; Paraguay 15; Thailand 15; Germany 14; Mexico 14; Suriname 13; Burundi 12; Philippines 12; Sierra Leone 12; Turkey 12; India 11; Chile 9; Malaysia 9; Serbia 9; Uruguay 9; Australia 8; Canada 8; Honduras 8; Hungary 8; Malawi 8; Netherlands 8; Senegal 8; Tanzania 8; Tunisia 8; Uganda 8; Benin 7; Burkina Faso 7; Czech Republic 7; Iran 7; Jamaica 7; Lebanon 7; Pakistan 7; Armenia 5; Belgium 5; Cyprus 5; Indonesia 5; Kazakhstan 5; Liberia 5; Nicaragua 5; Sweden 5; Switzerland 5; Uzbekistan 5; Finland 4; Greece 4; Hong Kong 4; Mali 4; Mauritius 4; Moldova 4; Nepal 4; Sao Tome and Principe 4; Trinidad and Tobago 4; Zambia 4; Austria 3; Congo (Brazzaville) 3; Egypt 3; Estonia 3; Gambia, The 3; [file JIA2-21-e25123-s001.docx]

# Additional file 1

| **Country** | **Committee** | **Number** |
| --- | --- | --- |
| Belgium (Antwerp) | Institute of Tropical Medicine, Institutional Review Board | 911/13 |
| Belgium (Brussels) | Comité local d’éthique hospitalier, Centre Hospitalier Universitaire Saint-Pierre | B076201215754 |
| Belgium (Gent) | Universitair Ziekenhuis Gent, Commissie voor Medische Ethiek | B076201215754 |
| Belgium (Liège) | Comité local d’éthique Hospitalo-Facultaire Universitaire de Liège | B707201318791 |
| Germany (Bonn) | Rheinische Friedrich-Wilhelms-Universität Bonn, Medizinische Fakultat Ethik-Kommission | 008/14 |
| Greece | National and Kapodistrian University of Athens Institutional Review Board | 6/3/2013a(# 6313) |
| Italy | Istituto Nazionale per le Malattie Infettive “Lazzoro Spallanzani” | 22/02/2013a |
| The Netherlands | Universiteit van Amsterdam | 2013_137#C20131038 |
| Portugal | Centro Hospitalar de São João, EPE | 28/8/2013a |
| Portugal | Hospital Prof. Doutor Fernando Fonseca, EPE | 31/8/2013a |
| Portugal | Centro Hospitalar Lisboa Norte, EPE | 9/10/2013a |
| Portugal | Centro Hospitalar Lisboa Central, EPE | 11/7/2013a |
| Portugal | Centro Hospitalar de Setúbal, EPE | 21/1/2015a |
| Portugal | Comissão Nacional de Proteção de Dados (Portuguese Data Protection Authority), EPE | 14/10/2014a |
| Spain | Comité de Ética de la Investigación y del bienestar animal, Instituto de Salud Carlos III | CEI PI 01_2012-v2 |
| Switzerland | Kantonale Ethikkommission Bern | 024/13 |
| United Kingdom | London-Bentham Research Ethics Committee | 11/LO/1600 |

^a^Date of approval letter.

# Additional file 2


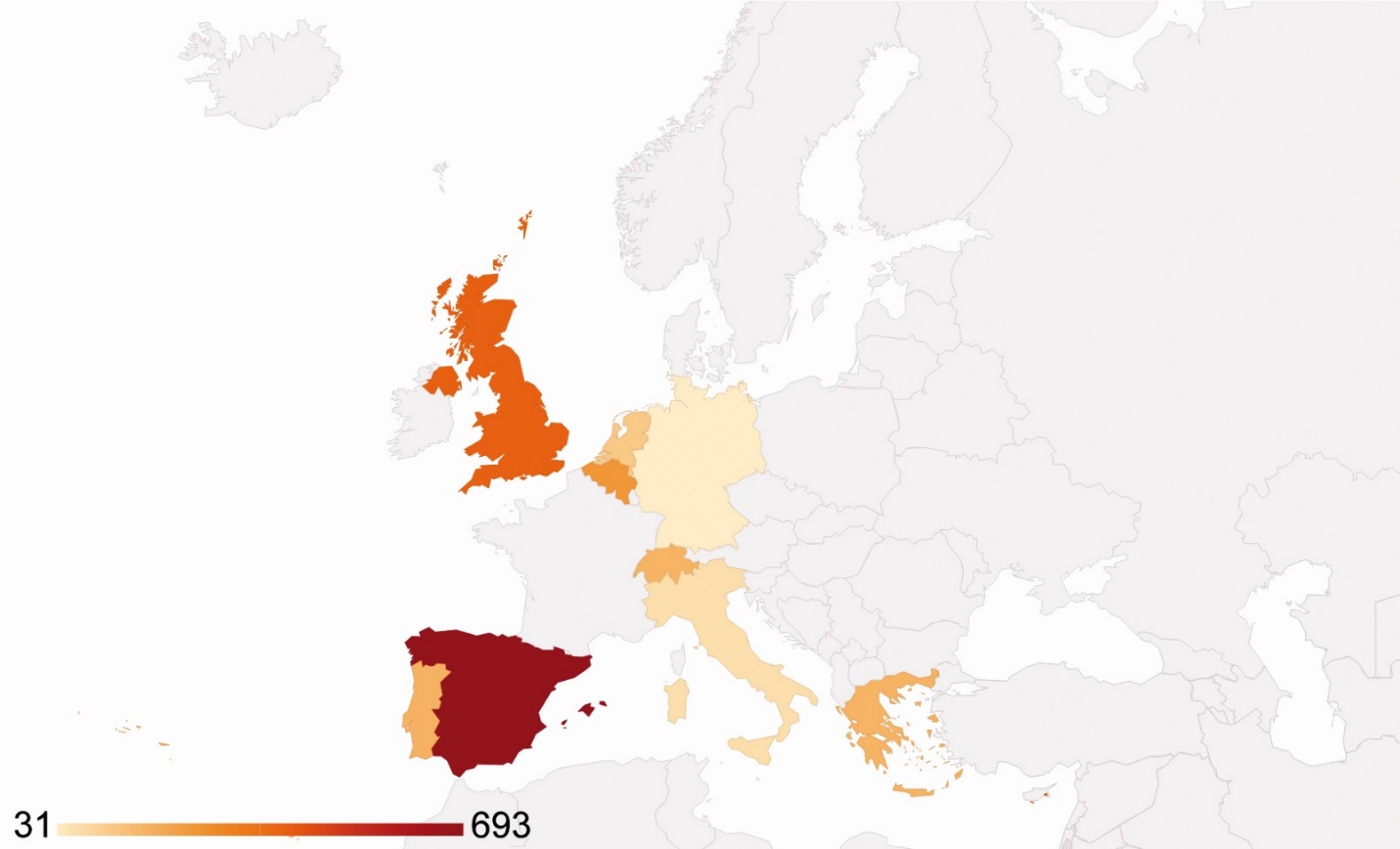


Figure 1: Current country of residence of male and female respondents to the aMASE Clinic Survey. N=2093 from 57 clinic sites (min, max patients 1, 148): Belgium 255, 4 clinics (27,148; Germany 31, 2 clinics (14, 17; Greece 175, 8 clinics (1,60) Italy 63, 2 clinics (20,43); Netherlands 119, 3clinics (28,51); Portugal 179, 7 clinics (5,54); Spain 693, 18 clinics (9,141); Switzerland 177, 6 clinics (5,42); United Kingdom 401, 7 clinics (21,106)


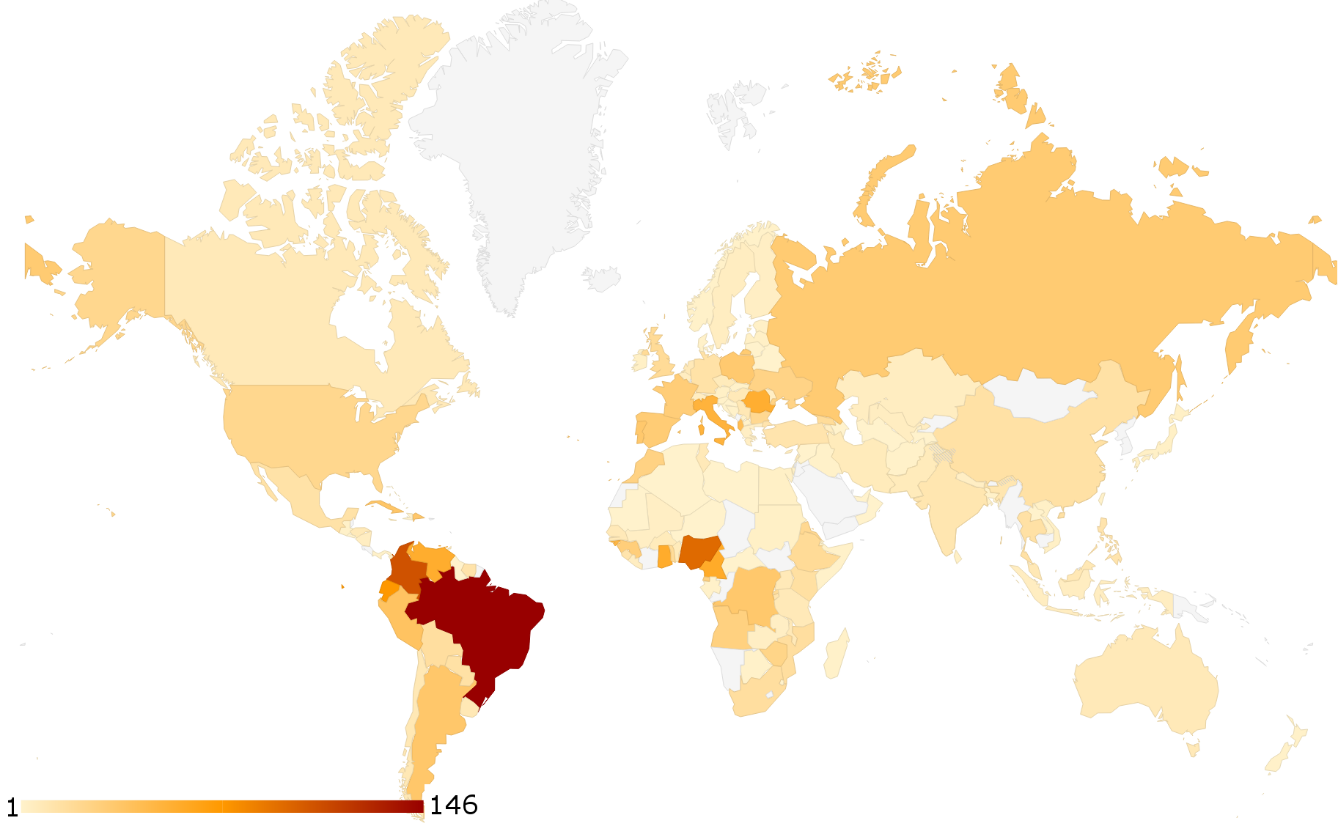


Figure 2: Country of birth of male and female respondents to the aMASE clinic survey. N=2093 from 152 countries. Brazil 146; Colombia 107; Nigeria 96; Ecuador 74; Cameroon 59; Ghana 58; Venezuela 57; Romania 56; Italy 52; Guinea-Bissau 49; Albania 43; Peru 39; Cuba 37; Argentina 36; Dominican Republic 36; Congo (Kinshasa) 35; Portugal 35; Poland 34; Russia 33; Spain 32; France 31; Guinea 30; Angola 28; Equatorial Guinea 27; Morocco 27; Ukraine 26; Cote d'Ivoire 25; Zimbabwe 25; Cape Verde 24; United States of America 23; Bulgaria 22; Eritrea 22; Ethiopia 20; United Kingdom 20; Georgia 19; Rwanda 19; Togo 19; Bolivia 17; Mozambique 17; South Africa 17; Kenya 16; China 15; Paraguay 15; Thailand 15; Germany 14; Mexico 14; Suriname 13; Burundi 12; Philippines 12; Sierra Leone 12; Turkey 12; India 11; Chile 9; Malaysia 9; Serbia 9; Uruguay 9; Australia 8; Canada 8; Honduras 8; Hungary 8; Malawi 8; Netherlands 8; Senegal 8; Tanzania 8; Tunisia 8; Uganda 8; Benin 7; Burkina Faso 7; Czech Republic 7; Iran 7; Jamaica 7; Lebanon 7; Pakistan 7; Armenia 5; Belgium 5; Cyprus 5; Indonesia 5; Kazakhstan 5; Liberia 5; Nicaragua 5; Sweden 5; Switzerland 5; Uzbekistan 5; Finland 4; Greece 4; Hong Kong 4; Mali 4; Mauritius 4; Moldova 4; Nepal 4; Sao Tome and Principe 4; Trinidad and Tobago 4; Zambia 4; Austria 3; Congo (Brazzaville) 3; Egypt 3; Estonia 3; Gambia, The 3; Ireland 3; Israel 3; Japan 3; Latvia 3; Netherlands Antilles 3; Sri Lanka 3; Sudan 3; Vietnam 3; Afghanistan 2; Antigua and Barbuda 2; Bangladesh 2; Bosnia and Herzegovina 2; Botswana 2; Denmark 2; Gabon 2; Guatemala 2; Iraq 2; Kosovo 2; Macedonia 2; Madagascar 2; New Zealand 2; Norway 2; Panama 2; Seychelles 2; Slovakia 2; Slovenia 2; Somalia 2; Taiwan 2; Algeria 1; Azerbaijan 1; Bahamas, The 1; Barbados 1; Belarus 1; Burma 1; Central African Republic 1; Comoros 1; Croatia 1; Djibouti 1; Dominica 1; El Salvador 1; Guyana 1; Haiti 1; Laos 1; Libya 1; Lithuania 1; Mauritania 1; Niger 1; Oman 1; Swaziland 1; Syria 1; Tajikistan 1; Timor-Leste 1; Turkmenistan 1; United Arab Emirates 1

# Additional file 3

| Table 2: Sociodemographic characteristics of survey respondents, by gender (men separated by sexual orientation) | | | | |
| --- | --- | --- | --- | --- |
|  | **Women** | **Heterosexual Men** | **Gay/Bisexual Men** | **p-value** |
| Total number of respondents n(%) | 658 (31.4) | 446 (21.3) | 989 (47.3) |  |
| Median age in years (IQR) | 37 (30.9 – 44.6) | 41 (34.3 – 48.4) | 35 (29.4 – 41.6) |  |
| Region of birth |  |  |  | <0.001 |
| *Africa* | 415 (63.1) | 254 (57.0) | 73 (7.4) |  |
| *Latin America/Caribbean* | 120 (18.2) | 72 (16.1) | 456 (46.1) |  |
| *Rest of World* | 32 (4.9) | 39 (8.7) | 146 (14.8) |  |
| *Europe* | 91 (13.8) | 81 (18.2) | 314 (31.7) |  |
| Mean age in years at migration (SD) | 29.3 (9.9) | 30.1 (10.0) | 26.3 (8.7) | <0.001 |
| Median years since migration (IQR) | 7 (4.1 – 12.7) | 10 (6.1 – 15.0) | 9 (4.8 – 13.9) |  |
| Ethnicity (n=1881) |  |  |  | <0.001 |
| *Black African/Caribbean* | 334 (59.5) | 205 (51.8) | 51 (5.5) |  |
| *White European* | 92 (16.4) | 69 (17.4) | 296 (32.0) |  |
| *Latin American/Hispanic* | 39 (7.0) | 26 (6.6) | 177 (19.2) |  |
| *Mixed Ethnicity* | 44 (7.8) | 30 (7.6) | 204 (22.1) |  |
| *Other* | 52 (9.3) | 66 (16.7) | 196 (21.2) |  |
| Education: Upper secondary or more | 322 (48.9) | 228 (51.1) | 802 (81.1) | <0.001 |
| Employment Status: working full/part time | 276 (41.9) | 217 (48.7) | 666 (67.3) | <0.001 |
| Relationship status |  |  |  | 0.0054 |
| *Married/Cohabitating* | 273 (41.5) | 195 (43.7) | 352 (35.6) |  |
| *Single* | 302 (45.9) | 170 (38.1) | 513 (51.9) |  |
| *Living apart relationship/marriage* | 83 (12.6) | 81 (18.2) | 124 (12.5) |  |
| Has children | 474 (72.6) | 301 (69.2) | 97 (9.9) | <0.001 |
| Religion of those who attend services (n=1165) |  |  |  | <0.001 |
| *Christian (All denominations)* | 428 (85.8) | 235 (76.1) | 306 (85.7) |  |
| *Muslim* | 48 (9.6) | 67 (21.7) | 13 (3.6) |  |
| *Other* | 23 (4.6) | 7 (2.3) | 38 (10.6) |  |
| Sexual Orientation (n=2076) |  |  |  | <0.001 |
| *Gay / Lesbian* | 12 (1.8) | 0 (0.0) | 843 (85.2) |  |
| *Heterosexual* | 616 (94.5) | 417 (95.9) | 0 (0.0) |  |
| *Bisexual* | 14 (2.1) | 0 (0.0) | 146 (14.8) |  |
| *Other* | 10 (1.5) | 18 (4.1) | 0 (0.0) |  |
| Monthly income compared to national minimum wage (n=1975) |  |  |  | <0.001 |
| *More or a lot more* | 65 (10.6) | 60 (14.3) | 430 (45.6) |  |
| *About the same* | 82 (13.4) | 70 (16.7) | 167 (17.7) |  |
| *Less than minimum wage* | 215 (35.0) | 126 (30.1) | 189 (20.1) |  |
| *Own wage not earned* | 236 (38.4) | 148 (35.3) | 140 (14.7) |  |
| *Not Known* | 16 (2.6) | 15 (3.6) | 16 (1.7) |  |
| Moderate /severe household hunger in past 4 weeks (n=2006) | 136 (21.8) | 112 (26.8) | 124 (12.8) | <0.001 |
| Immigration Status (n=2078) |  |  |  | <0.001 |
| *Permanent residency* | 335 (51.5) | 258 (58.4) | 777 (78.8) |  |
| *Temporary residency* | 238 (36.6) | 147 (33.3) | 152 (15.4) |  |
| *Asylum seeker/Refugee status* | 77 (11.8) | 37 (8.4) | 57 (5.8) |  |
| *Unknown* | 58 (8.9) | 23 (2.2) | 48 (4.9) |  |
| Travelled back to country of birth in past year | 191 (29.0) | 133 (29.8) | 497 (50.3) | <0.001 |
| Ever used a needle to inject drugs (n=2081) | 11 (1.7) | 34 (7.7) | 52 (5.3) | 0.001 |
| Ever received money, food or drugs for sex (n=2016) | 54 (8.5) | 27 (6.4) | 155 (16.2) | <0.001 |
| Previously paid money for sex (n=2035) | 5 (0.8) | 145 (34.5) | 136 (14.0) | <0.001 |

Data are n (%), median (Inter-quartile range), or mean (Standard Deviation). N=2093 unless otherwise stated.

| Table 3: Characteristics of survey respondents by gender (men separated by sexual orientation) at time of diagnosis | | | |
| --- | --- | --- | --- |
|  | **Women** | **Heterosexual Men** | **Gay/Bisexual Men** |
| Median age in years at diagnosis (IQR) | 34 (28.4 – 41.8) | 38 (31.7 – 45.7) | 34 (28.4 – 41.8) |
| Median CD4 cell count at diagnosis (IQR) (n=1815)* | 277 (124 – 438) | 240 (85 – 409) | 450 (276 – 639) |
| Late diagnosis (n=1815)* |  |  |  |
| *Diagnosed <350 cells mm^3^* | 293 (50.0) | 227 (56.9) | 248 (29.8) |
| *Diagnosed <200 cells mm^3^* | 173 (29.5) | 148 (37.1) | 110 (13.3) |
| Median years between migration and diagnosis (n=1859) | 5 (1 – 10) | 8 (3 – 13) | 7 (3 – 12) |
| Country of diagnosis (n=2081) |  |  |  |
| *Current country of residence* | 598 (91.7) | 416 (93.9) | 864 (87.6) |
| *Country of birth* | 37 (5.7) | 18 (4.1) | 86 (8.7) |
| *Other country* | 17 (2.6) | 9 (2.0) | 36 (3.7) |
| Diagnosed in Europe (n=1596^)&^ | 525 (93.4) | 341 (94.2) | 612 (91.1) |
| AIDS defining illness within 3 months of diagnosis (n=1997) | 101 (16.0) | 86 (20.5) | 63 (6.7) |
| Previous self-reported negative HIV test (n=2028) ** | 294 (46.7) | 183 (43.4) | 801 (82.0) |
| Time between negative test and diagnosis (n=1315)* |  |  |  |
| < 1 year | 21 (6.8) | 18 (9.5) | 181 (22.2) |
| < 2 years | 41 (13.3) | 39 (20.5) | 255 (31.3) |
| ≥ 2 years | 247 (79.9) | 133 (70.0) | 380 (46.6) |
| Attended health services in the 2 years prior to diagnosis (n=1878)* | 423 (70.7) | 310 (74.5) | 717 (83.0) |
| Testing discussed (n=1448)* |  |  |  |
| *Yes* | 105 (24.8) | 81 (26.3) | 279 (38.9) |
| *No* | 288 (68.1) | 207 (67.2) | 389 (54.3) |
| *Cannot recall* | 30 (7.1) | 20 (6.5) | 49 (6.8) |
| *Place where offered HIV test before diagnosis** |  |  |  |
| *Antenatal (n=55)* | 26 (49) | - | - |
| *Inpatient (n=255)* | 24 (29.6) | 13 (22.4) | 24 (27.9) |
| *Emergency (n=322)* | 5 (5.7) | 5 (6.9) | 13 (8.0) |
| *Sexual health clinic or HIV testing clinic (n=257)* | 14 (66.7) | 16 (69.6) | 156 (73.2) |
| *Outpatient (n=317)* | 15 (15.2) | 15 (23.8) | 35 (22.6) |
| *Dentist (n=431)* | 2 (2.0) | 6 (6.4) | 14 (5.9) |
| *GP/ Family Doctor (n=690)* | 23 (11.4) | 32 (21.3) | 97 (28.6) |
| *Other services (n=928)* | 15 (5.0) | 14 (6.3) | 15 (3.7) |
| Place of diagnosis (n=1878) * |  |  |  |
| *Antenatal service* | 74 (12.4) | 3 (0.7) | 3 (0.3) |
| *Hospital service e.g. Emergency / Inpatient / Outpatient* | 240 (40.1) | 196 (47.2) | 171 (19.8) |
| *Sexual health clinic or HIV testing clinic* | 75 (12.5) | 66 (15.9) | 376 (43.5) |
| *GP/ Family Doctor* | 105 (17.5) | 95 (22.9) | 201 (23.3) |
| *Private clinic* | 17 (2.8) | 7 (1.7) | 43 (5.0) |
| *Other* | 88 (14.7) | 48 (11.6) | 70 (8.1) |
| Tested because unwell/health problems | 261 (39.7) | 230 (51.6) | 256 (25.9) |

*Those diagnosed in current country of residence only; **Data missing from self-report supplemented from clinic records; Data are n (%), median (Inter-quartile range), or mean (Standard Deviation). N=2093 unless otherwise stated. & Non-European born

| Table 4: HIV treatment characteristics of aMASE clinic survey respondents, by gender (men separated by sexual orientation) | | | |
| --- | --- | --- | --- |
|  | **Women** | **Heterosexual Men** | **Gay/Bisexual Men** |
| Most recent CD4 cell count (cells mm3; n=2011) |  |  |  |
| *<200* | 67 (10.4) | 62 (14.4) | 35 (3.7) |
| *200-349* | 82 (12.8) | 87 (20.2) | 88 (9.4) |
| *350+* | 494 (76.8) | 282 (65.4) | 814 (86.9) |
| Undetectable viral load (<50 copies/ml; n=1540) | 409 (77.2) | 290 (75.9) | 489 (77.9) |
| Currently not on HIV treatment (n=2090) | 105 (16.0) | 40 (9.0) | 312 (31.6) |
| Reason not on HIV treatment *(n=457)* |  |  |  |
| *Doctor's advice or newly diagnosed* | 90 (85.7) | 33 (82.5) | 276 (88.5) |
| *High cost or otherwise inaccessible* | 3 (2.9) | 0 (0.0) | 15 (4.8) |
| *Fear of side effects or other difficulties taking medication* | 9 (8.6) | 5 (12.5) | 25 (8.0) |
| *Other reason* | 7 (6.7) | 3 (7.5) | 16 (5.1) |
| Access to primary care (n=2076) | 552 (85.1) | 369 (83.5) | 833 (84.6) |
| HIV treatment and care payments (n=972) |  |  |  |
| *Government/State* | 244 (78.2) | 162 (78.6) | 319 (70.3) |
| *Private Health Insurance/Self pay* | 21 (6.7) | 18 (8.7) | 84 (18.5) |
| *Do not Know* | 35 (11.2) | 19 (9.2) | 33 (7.3) |
| *Other/Charity* | 12 (3.8) | 7 (3.4) | 18 (4.0) |
| Experienced difficulties with health service in CCOR (n=2093) | 211 (32.3) | 132 (29.9) | 272 (27.7) |
| *No GP/Health card/insurance (n=628)* | 33 (15.3) | 18 (13.1) | 58 (20.9) |
| *Unclear of rights to access medical care (n=629)* | 43 (19.9) | 35 (25.5) | 70 (25.3) |
| *Inconvenient clinic hours (n=628)* | 11 (5.1) | 10 (7.3) | 46 (16.6) |
| *Long waiting times for an appointment/in the clinic (n=628)* | 72 (33.3) | 29 (21.2) | 111 (40.1) |
| *Does not trust the GP confidentiality (n=628)* | 48 (22.2) | 31 (22.6) | 37 (13.4) |
| *Difficulty communicating with staff because of language differences (n=628)* | 55 (25.5) | 38 (27.7) | 38 (13.7) |
| *Difficulty negotiating healthcare system (e.g. finding GP, payment, travel) (n=629)* | 22 (10.2) | 13 (9.5) | 31 (11.2) |
| *Poor quality service or experienced prejudice* | 8 (3.7) | 3 (2.2) | 4 (1.4) |
| Missed clinical appointments because of travel expenses (n=2071) |  |  |  |
| Yes | 77 (11.9) | 66 (15.1) | 68 (6.9) |
| No | 544 (83.8) | 350 (79.9) | 881 (89.5) |
| I do not have to pay for travel to the clinic | 28 (4.3) | 22 (5.0) | 35 (3.6) |
| Delayed/forwent medication because of prescription costs (n=2078) |  |  |  |
| *Yes* | 54 (8.3) | 39 (8.8) | 48 (4.9) |
| *No* | 318 (48.9) | 216 (48.9) | 486 (49.3) |
| *Does not pay for medication* | 230 (35.4) | 169 (38.2) | 287 (29.1) |
| *Not taking medication* | 48 (7.4) | 18 (4.1) | 165 (16.7) |

Data are n (%), median (Inter-quartile range), or mean (Standard Deviation). N=2093 unless otherwise stated. CCOR=Current Country of Residence OR= odds ratio. AOR=adjusted odds ratio. 95% CI= 95% Confidence Interval. ART=Antiretroviral Therapy.
